# Serum BTP concentrations are not affected by hepatic dysfunction

**DOI:** 10.1186/s12882-018-0881-x

**Published:** 2018-04-13

**Authors:** Debarati Chakraborty, Ayub Akbari, Greg A. Knoll, Jennifer A. Flemming, Catherine Lowe, Shareef Akbari, Christine A. White

**Affiliations:** 1 Division of Nephrology, Department of Medicine, Queen’s University, Etherington Hall, 94 Stuart Street, Kingston, ON K7L 2N6 Canada; 2 Division of Nephrology, Department of Medicine, University of Ottawa, Ottawa, ON Canada; 3 Kidney Research Centre, Ottawa Health Research Institute, Ottawa, ON Canada; 4 Clinical Epidemiology Program, Ottawa Health Research Institute, Ottawa, ON Canada; 5 Division of Gastroenterology, Department of Medicine, Queen’s University, Kingston, ON Canada; 6 Department of Public Health Sciences, Queen’s University, Kingston, ON Canada

**Keywords:** Beta trace protein, Cirrhosis, Creatinine, Cystatin C, Glomerular filtration rate

## Abstract

**Background:**

Beta Trace Protein (BTP) is a promising marker of glomerular filtration rate (GFR). Equations to estimate GFR using BTP have been proposed. Very little is known about BTP’s production and metabolism. It has been hypothesized that the liver metabolizes certain BTP isoforms. As such, hepatic dysfunction may influence serum levels independently of GFR. This would impact on the accuracy and precision of GFR estimates using BTP. The purpose of this study was to assess the impact of cirrhosis on serum BTP concentrations.

**Methods:**

BTP, cystatin C (cysC) and creatinine (Cr) were measured in 99 cirrhotic subjects and in matched controls. BTP/cysC and Cr/cysC ratios were compared between cases and controls. This was repeated after stratification by Child Pugh category. Comparisons of ratios between Child Pugh category A and combined B and C case subjects were also performed.

**Results:**

There were no differences in BTP/cysC ratios between cases and controls for the entire cohort (0.80 vs 0.79) or for any of the Child Pugh categories (*p* > 0.10). There were significant differences between cases (1.09) and controls (0.73) for the BTP/Cr ratios (*p* < 0.001). The BTP/Cr ratio was higher in those with more advanced cirrhosis as compared to those with less severe cirrhosis (1.20 vs 1.03, *p* < 0.01). There were no differences in BTP/cysC ratios between those with less severe and more advanced cirrhosis (*p* = 0.25).

**Conclusions:**

This study suggests that hepatic dysfunction does not influence serum BTP levels and argues against a significant role for the liver in BTP metabolism. Confirmation in a larger group of patients with advanced cirrhosis is required.

## Background

Beta Trace Protein (BTP) is a low molecular weight glycoprotein and a novel endogenous marker of glomerular filtration rate (GFR) [1]. First described in 1961 [2], it was noted to be increased in the serum of patients with renal disease in 1997 [3]. Subsequent investigations have examined the utility of BTP as a marker of GFR in a variety of patient populations [4–9]. Some evidence suggests that it is more sensitive than creatinine (Cr) at detecting early changes in GFR [10, 11]. Several BTP-based GFR estimation equations have been proposed [8, 12–15]. These however have generally been found to less accurate and more imprecise than equations containing Cr and cystatin C (cysC) suggesting a greater impact of non-GFR determinants on serum BTP concentrations as compared to the other two filtration markers [8, 16]. Non-GFR determinants of BTP concentrations thus further identified include serum albumin concentration, gender, urine protein excretion and weight [17, 18].

Unlike Cr, very little is known about the origin and metabolism of BTP. BTP is a heterogeneous glycoprotein with multiple isoforms and is present in various fluid compartments including blood, urine and cerebral spinal fluid (CSF) [1, 3]. Smaller non-sialyzed isoforms predominate in the CSF whereas the larger sialylated isoforms predominate in the serum and urine [3]. While a number of cell types have been demonstrated to produce BTP, the origin of serum BTP is unclear [1]. It has been hypothesized to result from diffusion from the CSF based on the glycosylation patterns of BTP glycoforms which are most typical for CSF as supposed to hepatic glycoproteins [2, 3]. In an animal study, intra-thecally administered recombinant BTP was recovered from serum lending support for a CNS origin [19]. It has been further hypothesized that the non-sialylated “brain” glycoforms are then rapidly eliminated from blood by the liver leaving a predominance of the “blood/urine” sialylated glycoforms [3].

The impact of hepatic dysfunction on serum BTP concentrations has never been investigated. Its effect on serum Cr concentrations is well recognized with depressed serum concentrations due to decreased hepatic synthesis of its precursor creatine, malnutrition and muscle wasting [20, 21]. These factors significantly hamper the assessment of kidney function in cirrhotics [20–22]. If the above described hypotheses are correct, non-sialyated BTP isoforms would accumulate in the circulation in the setting of hepatic dysfunction, leading to increased serum levels and reduced accuracy of BTP-based GFR estimates. The aim of this study was to determine whether patients with hepatic dysfunction have higher than expected serum BTP concentrations due to reduced hepatic clearance of the non-sialyzed isoforms.

## Methods

This case-control study received institutional ethics approval and was conducted between June–October 2014 at the academic hospitals of Queen’s University Kingston, ON, Canada. Case subjects were recruited in the tertiary care Liver Clinic staffed by two subspecialty trained hepatologists and included if they had a diagnosis of cirrhosis and excluded if they were dialysis dependent or had known acute kidney injury. The diagnosis of cirrhosis was confirmed by the hepatologists according to standard clinical criteria of either 1) cirrhosis on liver biopsy; 2) evidence of portal hypertension or hepatic decompensation in the form of ascites, esophageal varices or hepatic encephalopathy or; 3) Non-invasive testing (FibroTest© or FibroScan©) estimating F4 fibrosis in an individual with known chronic liver disease. Basic demographic, clinical and laboratory data were collected including diabetes status, etiology of cirrhosis, presence of ascites or encephalopathy, INR, albumin and bilirubin. Child Pugh Class and MELD scores were calculated [23, 24]. The Child Pugh classification incorporates five variables (albumin, ascites, encephalopathy, INR) while the MELD score includes bilirubin, Cr, and INR (Table 1).

**Table 1:** Child Pugh and MELD scores

Child Pugh^a^			
Measure	1 point	2 points	3 points
Bilirubin, (mg/dl)	< 2	2–3	> 3
Albumin, g/dl	> 3.5	2.8–3.5	< 2.8
INR	< 1.7	1.71–2.30	> 2.30
Ascites	None	Mild	Moderate to severe
Hepatic encephalopathy	None	Grade I-II (or suppressed)	Grade III-IV (or refractory)
MELD score^b^			
(0.957 * log_e_(creatinine) + 0.378 * log_e_(bilirubin) + 1.120 * log_e_(INR) + 0.6431) *10			

^a^Class A (5,6 points), Class B (7–9 points), Class C (10–15 points)

^b^Creatinine and bilirubin in mg/dL, maximum creatinine concentration is 4.0 mg/dL

Control subjects consisted of patients attending renal clinics who had blood work done in clinic as part of routine clinical care. Exclusion criteria were known liver disease or cirrhosis. Renal patients were chosen as the control group because they have sufficient residual serum left after their routine biochemistry is performed to allow for study analyte measurement. Controls were matched 1:1 to case patients by age (per 10 year strata), gender and diabetes status as these variables are associated with cystatin C or BTP independently of GFR [18, 25].

Cystatin C (cysC), BTP (Siemen’s nephelometric assays) and Cr (Vitros Chemistry enzymatic assay) were measured at the Children’s Hospital of Eastern Ontario, ON, Canada. The BTP/cysC ratio was calculated for each subject. The BTP/cysC ratio was chosen in lieu of the BTP/Cr ratio due to the well-recognized inaccuracy of serum creatinine as a marker of GFR in the setting of hepatic dysfunction. Prior studies have shown a strong inverse relationship between inulin GFR and cysC in cirrhotic patients and a lack of impact of cirrhosis on serum cysC levels, suggesting that cysC is an acceptable surrogate for measured GFR [20, 26]. As the control group had known CKD, the BTP/cysC ratio was selected for the outcome instead of the BTP serum concentration in order to adjust for GFR. The difference in mean BTP/cysC ratio between the groups was compared using t-tests. This was repeated after subdividing patients by Child Pugh classification. Similar analysis was done using BTP/Cr. The BTP/cysC and BTP/Cr ratios of the combined Child Pugh B and C category cirrhotics were compared using t tests to those of the Child Pugh A category cirrhotic case subjects.

## Results

**Fig. 1:**
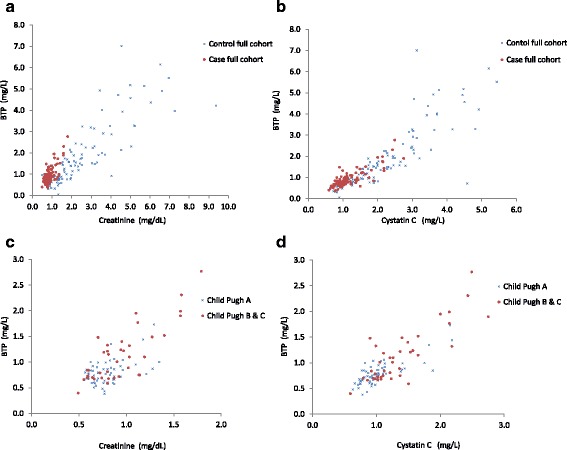
Scatterplots of analytes concentrations: (**a**) BTP (mg/L) and Creatinine (mg/dL) in full cohort cases and controls: (**b**) BTP (mg/L) and CysC (mg/dL) in full cohort cases and controls: (**c**) BTP (mg/L) and Creatinine (mg/dL) in Child Puch A and Child Pugh B&C; (**d**) BTP (mg/L) and Cystatin C (mg/L) in Child Puch A and Child Pugh B&C

A total of 99 case patients were recruited over the study period and were matched with 99 controls. Patient characteristics are shown in Table 2. As expected, case and control subjects were similar with respect to age, gender and diabetic status. Thirty one percent of both case and control groups were diabetic. The most common etiology of cirrhosis was hepatitis C (38%). The majority of case patients had compensated cirrhosis (61% Child Pugh A). Mean MELD score was 11.0 ± 3.8. Mean Cr was significantly higher in controls (2.59 vs 0.85 in cirrhotics, *p* < 0.001). BTP and cysC were also higher in the control groups. Scatterplots of the analyte concentrations (case vs controls and Child Pugh A vs Child Pugh B&C) are found in Fig. 1.

**Table 2:** Case and control subject characteristics

Characteristic	Case (*n* = 99)	Controls (n = 99)	*P* value
Male, N (%)	70 (71)	70 (71)	0.88
Age, (SD) years	59.4 (7.9)	60.4 (8.6)	0.40
Diabetes, N(%)	31 (31)	31 (31)	0.88
Cirrhosis etiology		Not applicable	
Hepatitis C	38 (38)		
Ethanol	21 (21)		
NASH	21 (21)		
Hepatitis B	2 (2)		
Autoimmune	2 (2)		
Other (PBC, NYD)	6 (6)		
Cr, mean (SD), 95% CI, mg/dL	0.85 (0.25)	2.59 (0.28)	< 0.001
	0.77–0.93	2.50–2.67	
BTP, mean (SD), 95% CI, mg/L	0.93 (0.41)	1.92 (1.4)	< 0.001
	0.85–1.01	1.84–2.00	
CysC, mean (SD), 95% CI, mg/L	1.17 (0.44)	2.24 (1.16)	< 0.001
	1.09–1.25	2.16–2.32	
Bilirubin		Not measured	
mean (SD), mg/dL	1.6 (1.4)		
< 2 mg/dL, N(%)	77 (78)		
2–3 mg/dL, N(%)	9 (9)		
> 3 mg/dL, N(%)	13 (13)		
Albumin		Not measured	
mean (SD), g/dL	3.4 (0.6)		
> 3.5 g/dL, N(%)	47 (48)		
2.8–3.5 g/dL, N(%)	42 (42)		
< 2.8 g/dL, N(%)	10 (10)		
INR		Not measured	
mean (SD)	1.3 (0.3)		
< 1.7, N(%)	90 (91)		
1.7–2.3, N(%)	8 (8)		
> 2.3, N(%)	1 (1)		
Ascites N(%)			
None,	64 (65)	99	
Mild,	33 (33)	0	
Moderate-Severe	2 (2)	0	
Encephalopathy, N(%)			
None	80 (81)	99	
Grade I-II	19 (19)	0	
Grade III-IV	0 (0)	0	
CHILD PUGH category N (%)			
A	60 (61)	Not applicable	
B	27 (27)		
C	12 (12)		
MELD score N (%)			
≤ 9.4	50 (47.6)	Not applicable	
9.5–19	51 (48.6)		
20–29	4 (3.8)

**Fig. 2:**
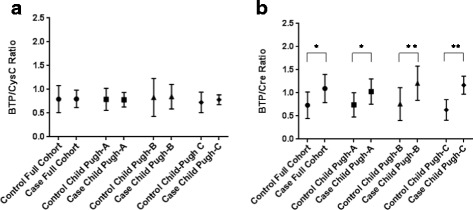
Mean BTP/cysC (**a**) and BTP/Cr ratios (**b**). Data are presented as the mean and standard deviation. There are no significant differences in BTP/cysC ratios between case and control groups (*p* > 0.10). The BTP/Cr ratios are significantly higher in the case groups as compared to the control groups for all three Child Pugh Categories. *p < 0.001,**p < 0.01

Figure 2 shows the ratios between the analytes. There were no significant differences between the control and case BTP/cysC ratios for the whole cohort or for any of the Child Pugh Classes. In comparison, there was a significantly higher BTP/Cr ratio in the case group as compared to the control group for the entire cohort and for each of the Child Pugh Classes.

The BTP/Cr ratio was higher for the combined Child Pugh B &C as compared to the Child Pugh A case subjects (1.20 vs 1.03, *p* < 0.01). There was no difference in the BTP/cysC ratios between the combined Child Pugh B &C and Child Pugh A cirrhotic groups (*p* = 0.25).

## Discussion

In this study, we have shown that the serum BTP concentration is not affected by the presence of cirrhosis. BTP/cysC ratios were similar to controls, even in those with the most advanced hepatic dysfunction. The lack of BTP/cysC ratio differences between the different Child Pugh categories further supports the absence of hepatic effect on BTP. This is in contrast to what is observed with serum Cr. The higher BTP/Cr ratios in the cirrhotic group and in cirrhotics with more advanced disease reflect a well-recognized decrease in Cr production in the setting of cirrhosis [20].

BTP for GFR prediction in the setting of cirrhosis was recently investigated by Mindikoglu et al. [22] GFR was measured by plasma clearance of iothalamate and serum was sampled for Cr, cysC, BTP and other analytes in 103 patients with cirrhosis. Regression analysis was used to develop a novel GFR prediction equation. The authors found that adding BTP to cycC and Cr did not confer any additional benefit for GFR prediction [22]. This study is somewhat hampered by the use of plasma clearance technique for GFR determination in most subjects. Plasma clearance protocols have been shown to be inaccurate in cirrhosis due to tracer sequestration into inaccessible compartments (ascites, peripheral edema) and are therefore not recommended for GFR assessment in this special population [27, 28]. Nonetheless, the study findings are consistent with what has been reported in other non-cirrhotic populations [16].

Our novel results argue against a significant role for the liver in the metabolism of BTP. Other hypothesis must therefore be explored to explain the relative amounts of different BTP isoforms that has been observed in different fluid compartments. An alternate potential explanation for our findings is that the anti-BTP antibodies used in Siemens’s nephelometric assay might not recognize epitopes present in the “brain type” isoforms. The Siemens assay uses polyclonal antibodies directed against human urinary BTP and therefore could theoretically not bind to brain type BTP isoforms. In this case, elevated brain BTP isoforms may exist but are simply not detectable.

Limitations include the use of CKD patients as the control group. This patient group was selected as control for pragmatic reasons: their co-morbidities are well documented, they are numerous and, unlike most other patient groups, they have routine phlebotomy with large volumes of residual plasma in which additional analytes can be measured. The absence of measured GFR is another limitation. Measuring GFR using exogenous markers is expensive and cumbersome particularly in the setting of cirrhosis which requires the more difficult urinary clearance methods to prevent GFR overestimation due to tracer clearance in extravascular compartments. Confirmation of study findings using a measured GFR would be beneficial. Finally, only 12% of the study population have advanced cirrhosis (12% Child Pugh C) which hinders extrapolation of study findings to those with advanced disease.

## Conclusions

It is well recognized that a number of factors (muscle mass, diet, hepatic function) influence serum Cr independently of GFR and these contribute significantly to the difficulties in accurately assessing GFR using Cr [22, 29]. This study suggests that, unlike serum Cr, serum BTP concentrations are independent of hepatic dysfunction. Additional studies exploring any incremental benefit of adding BTP to the existing panel of endogenous GFR markers in cirrhosis are needed. In addition, further study of other non-GFR dependent factors which may influence serum BTP concentration levels is required to allow for a better understanding of how to best incorporate BTP into clinical care.

## Abbreviations

BTP: Beta trace protein
CKD: Chronic kidney disease
Cr: Creatinine
CysC: Cystatin C
GFR: Glomerular filtration rate
MELD: Model for end-stage liver disease
